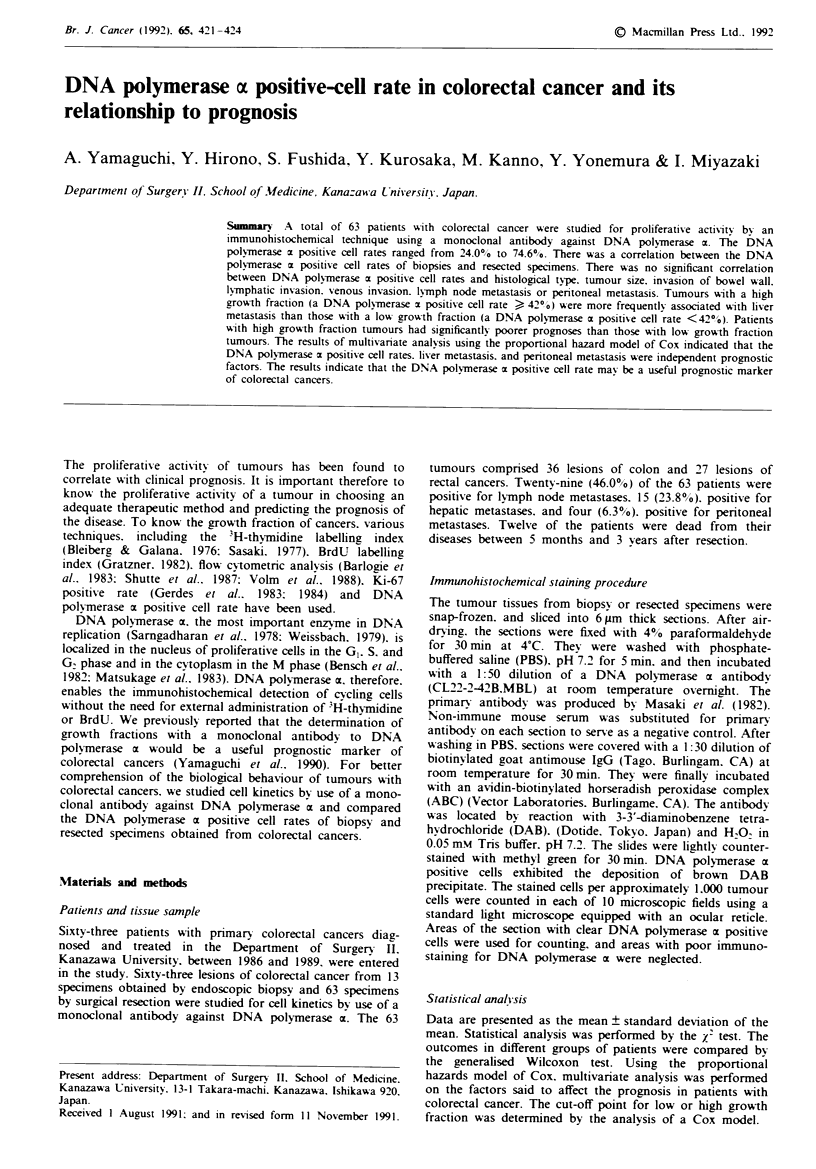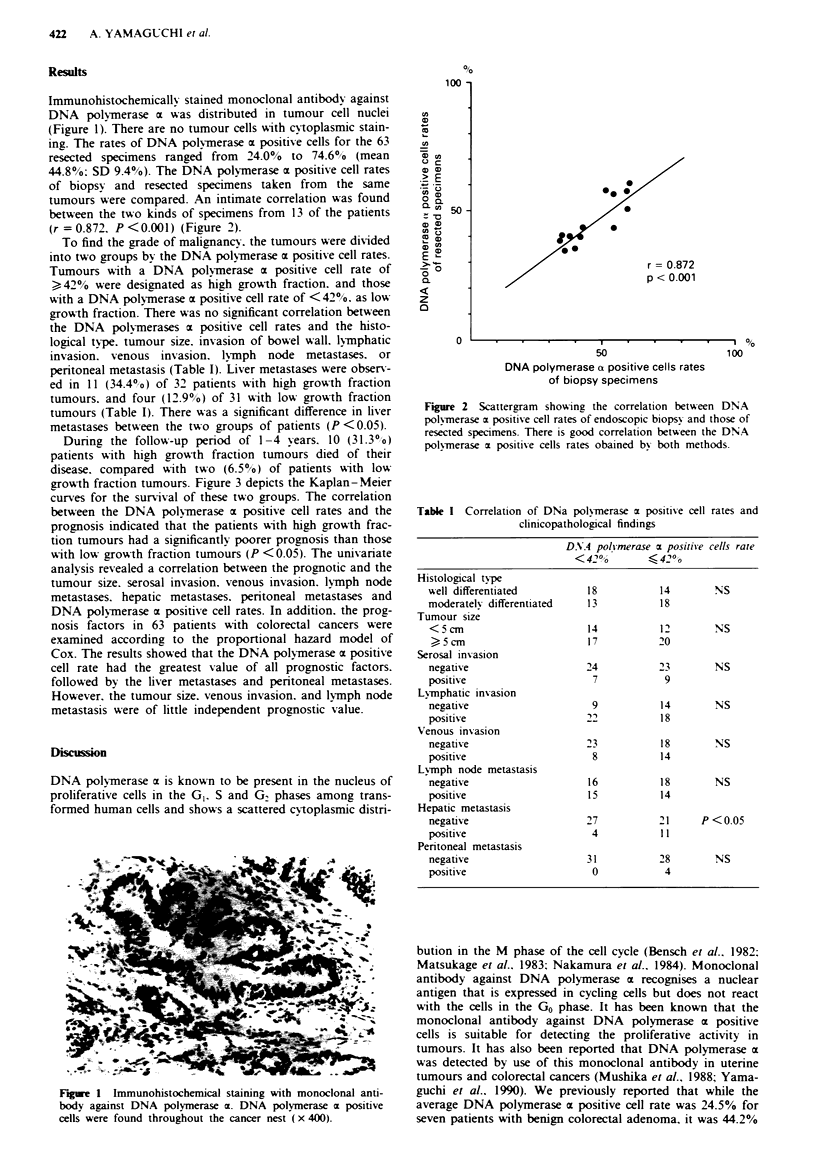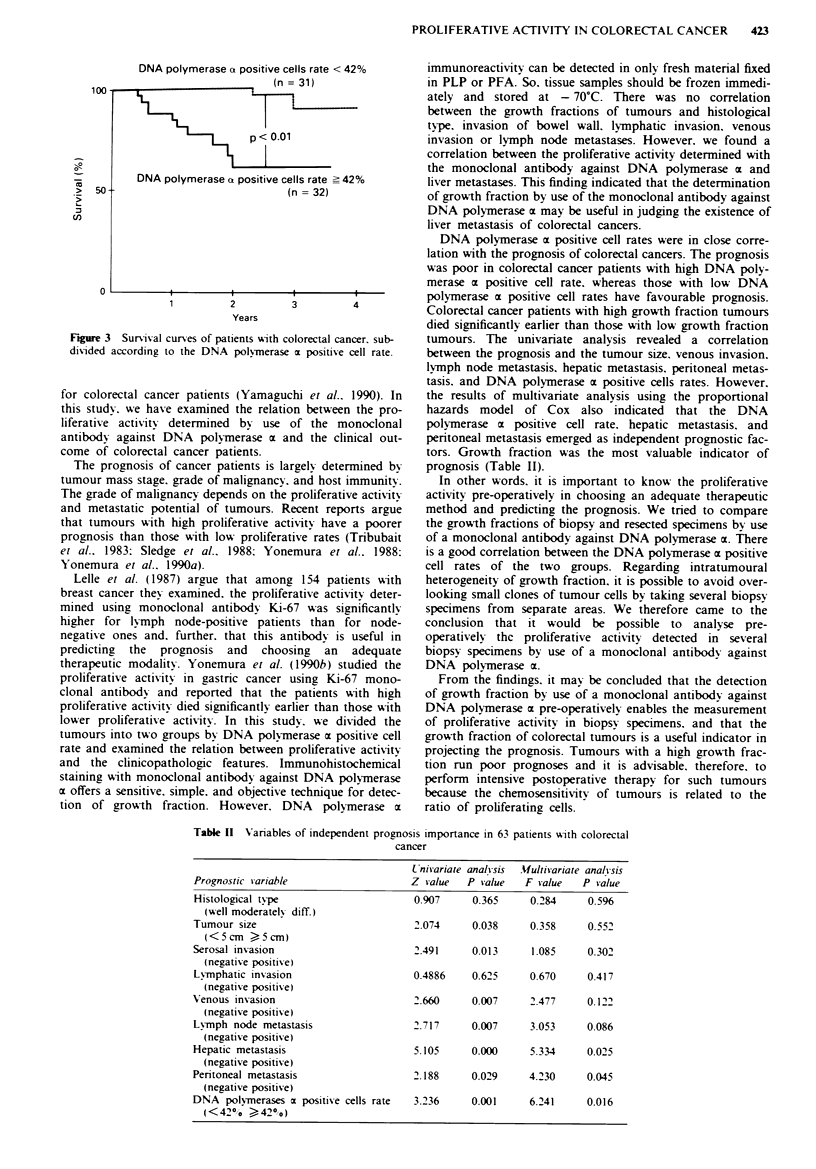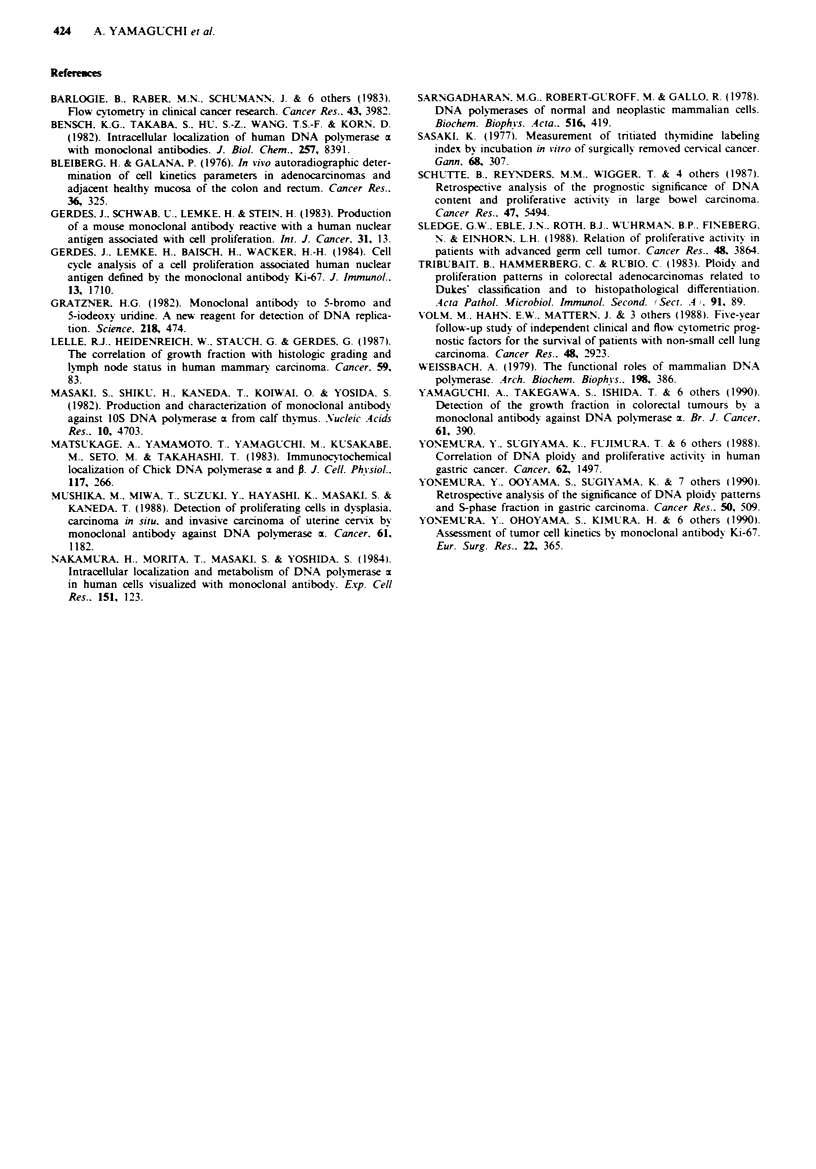# DNA polymerase alpha positive-cell rate in colorectal cancer and its relationship to prognosis.

**DOI:** 10.1038/bjc.1992.86

**Published:** 1992-03

**Authors:** A. Yamaguchi, Y. Hirono, S. Fushida, Y. Kurosaka, M. Kanno, Y. Yonemura, I. Miyazaki

**Affiliations:** Department of Surgery II, School of Medicine, Kanazawa University, Japan.

## Abstract

**Images:**


Br. J. Cancer (1992), 65, 421-424 © Macmillan Press Ltd., 1992

### DNA polymerase α positive-cell rate in colorectal cancer and its relationship to prognosis

A. Yamaguchi, Y. Hirono, S. Fushida, Y. Kurosaka, M. Kanno, Y. Yonemura & I. Miyazaki

*Department of Surgery II, School of Medicine, Kanazawa University, Japan.*

**Summary** A total of 63 patients with colorectal cancer were studied for proliferative activity by an immunohistochemical technique using a monoclonal antibody against DNA polymerase α. The DNA polymerase α positive cell rates ranged from 24.0% to 74.6%. There was a correlation between the DNA polymerase α positive cell rates of biopsies and resected specimens. There was no significant correlation between DNA polymerase α positive cell rates and histological type, tumour size, invasion of bowel wall, lymphatic invasion, venous invasion, lymph node metastasis or peritoneal metastasis. Tumours with a high growth fraction (a DNA polymerase α positive cell rate ⩾42%) were more frequently associated with liver metastasis than those with a low growth fraction (a DNA polymerase α positive cell rate <42%). Patients with high growth fraction tumours had significantly poorer prognoses than those with low growth fraction tumours. The results of multivariate analysis using the proportional hazard model of Cox indicated that the DNA polymerase α positive cell rates, liver metastasis, and peritoneal metastasis were independent prognostic factors. The results indicate that the DNA polymerase α positive cell rate may be a useful prognostic marker of colorectal cancers.

The proliferative activity of tumours has been found to correlate with clinical prognosis. It is important therefore to know the proliferative activity of a tumour in choosing an adequate therapeutic method and predicting the prognosis of the disease. To know the growth fraction of cancers, various techniques, including the $^{3}$H-thymidine labelling index (Bleiberg & Galana, 1976; Sasaki, 1977), BrdU labelling index (Gratzner, 1982), flow cytometric analysis (Barlogie *et al.*, 1983; Shutte *et al.*, 1987; Volm *et al.*, 1988), Ki-67 positive rate (Gerdes *et al.*, 1983; 1984) and DNA polymerase α positive cell rate have been used.

DNA polymerase α, the most important enzyme in DNA replication (Sarngadharan *et al.*, 1978; Weissbach, 1979), is localized in the nucleus of proliferative cells in the $G_1$, S, and $G_2$ phase and in the cytoplasm in the M phase (Bensch *et al.*, 1982; Matsukage *et al.*, 1983). DNA polymerase α, therefore, enables the immunohistochemical detection of cycling cells without the need for external administration of $^{3}$H-thymidine or BrdU. We previously reported that the determination of growth fractions with a monoclonal antibody to DNA polymerase α would be a useful prognostic marker of colorectal cancers (Yamaguchi *et al.*, 1990). For better comprehension of the biological behaviour of tumours with colorectal cancers, we studied cell kinetics by use of a monoclonal antibody against DNA polymerase α and compared the DNA polymerase α positive cell rates of biopsy and resected specimens obtained from colorectal cancers.

### Materials and methods

### *Patients and tissue sample*

Sixty-three patients with primary colorectal cancers diagnosed and treated in the Department of Surgery II, Kanazawa University, between 1986 and 1989, were entered in the study. Sixty-three lesions of colorectal cancer from 13 specimens obtained by endoscopic biopsy and 63 specimens by surgical resection were studied for cell kinetics by use of a monoclonal antibody against DNA polymerase α. The 63 tumours comprised 36 lesions of colon and 27 lesions of rectal cancers. Twenty-nine (46.0%) of the 63 patients were positive for lymph node metastases, 15 (23.8%), positive for hepatic metastases, and four (6.3%), positive for peritoneal metastases. Twelve of the patients were dead from their diseases between 5 months and 3 years after resection.

### *Immunohistochemical staining procedure*

The tumour tissues from biopsy or resected specimens were snap-frozen, and sliced into 6 μm thick sections. After air-drying, the sections were fixed with 4% paraformaldehyde for 30 min at 4°C. They were washed with phosphate-buffered saline (PBS), pH 7.2 for 5 min, and then incubated with a 1:50 dilution of a DNA polymerase α antibody (CL22-2-42B,MBL) at room temperature overnight. The primary antibody was produced by Masaki *et al.* (1982). Non-immune mouse serum was substituted for primary antibody on each section to serve as a negative control. After washing in PBS, sections were covered with a 1:30 dilution of biotinylated goat antimouse IgG (Tago, Burlingam, CA) at room temperature for 30 min. They were finally incubated with an avidin-biotinylated horseradish peroxidase complex (ABC) (Vector Laboratories, Burlingame, CA). The antibody was located by reaction with 3-3′-diaminobenzene tetrahydrochloride (DAB), (Dotide, Tokyo, Japan) and $H_2O_2$ in 0.05 mM Tris buffer, pH 7.2. The slides were lightly counterstained with methyl green for 30 min. DNA polymerase α positive cells exhibited the deposition of brown DAB precipitate. The stained cells per approximately 1,000 tumour cells were counted in each of 10 microscopic fields using a standard light microscope equipped with an ocular reticle. Areas of the section with clear DNA polymerase α positive cells were used for counting, and areas with poor immunostaining for DNA polymerase α were neglected.

### *Statistical analysis*

Data are presented as the mean ± standard deviation of the mean. Statistical analysis was performed by the $\chi^2$ test. The outcomes in different groups of patients were compared by the generalised Wilcoxon test. Using the proportional hazards model of Cox, multivariate analysis was performed on the factors said to affect the prognosis in patients with colorectal cancer. The cut-off point for low or high growth fraction was determined by the analysis of a Cox model.

Present address: Department of Surgery II, School of Medicine, Kanazawa University, 13-1 Takara-machi, Kanazawa, Ishikawa 920, Japan.
Received 1 August 1991; and in revised form 11 November 1991.

### Results

Immunohistochemically stained monoclonal antibody against DNA polymerase α was distributed in tumour cell nuclei (Figure 1). There are no tumour cells with cytoplasmic staining. The rates of DNA polymerase α positive cells for the 63 resected specimens ranged from 24.0% to 74.6% (mean 44.8%; SD 9.4%). The DNA polymerase α positive cell rates of biopsy and resected specimens taken from the same tumours were compared. An intimate correlation was found between the two kinds of specimens from 13 of the patients ($r = 0.872$, $P < 0.001$) (Figure 2).

To find the grade of malignancy, the tumours were divided into two groups by the DNA polymerase α positive cell rates. Tumours with a DNA polymerase α positive cell rate of ⩾42% were designated as high growth fraction, and those with a DNA polymerase α positive cell rate of <42%, as low growth fraction. There was no significant correlation between the DNA polymerases α positive cell rates and the histological type, tumour size, invasion of bowel wall, lymphatic invasion, venous invasion, lymph node metastases, or peritoneal metastasis (Table I). Liver metastases were observed in 11 (34.4%) of 32 patients with high growth fraction tumours, and four (12.9%) of 31 with low growth fraction tumours (Table I). There was a significant difference in liver metastases between the two groups of patients ($P < 0.05$).

During the follow-up period of 1–4 years, 10 (31.3%) patients with high growth fraction tumours died of their disease, compared with two (6.5%) of patients with low growth fraction tumours. Figure 3 depicts the Kaplan–Meier curves for the survival of these two groups. The correlation between the DNA polymerase α positive cell rates and the prognosis indicated that the patients with high growth fraction tumours had a significantly poorer prognosis than those with low growth fraction tumours ($P < 0.05$). The univariate analysis revealed a correlation between the prognotic and the tumour size, serosal invasion, venous invasion, lymph node metastases, hepatic metastases, peritoneal metastases and DNA polymerase α positive cell rates. In addition, the prognosis factors in 63 patients with colorectal cancers were examined according to the proportional hazard model of Cox. The results showed that the DNA polymerase α positive cell rate had the greatest value of all prognostic factors, followed by the liver metastases and peritoneal metastases. However, the tumour size, venous invasion, and lymph node metastasis were of little independent prognostic value.

### Discussion

DNA polymerase α is known to be present in the nucleus of proliferative cells in the $G_1$, S and $G_2$ phases among transformed human cells and shows a scattered cytoplasmic distribution in the M phase of the cell cycle (Bensch *et al.*, 1982; Matsukage *et al.*, 1983; Nakamura *et al.*, 1984). Monoclonal antibody against DNA polymerase α recognises a nuclear antigen that is expressed in cycling cells but does not react with the cells in the $G_0$ phase. It has been known that the monoclonal antibody against DNA polymerase α positive cells is suitable for detecting the proliferative activity in tumours. It has also been reported that DNA polymerase α was detected by use of this monoclonal antibody in uterine tumours and colorectal cancers (Mushika *et al.*, 1988; Yamaguchi *et al.*, 1990). We previously reported that while the average DNA polymerase α positive cell rate was 24.5% for seven patients with benign colorectal adenoma, it was 44.2%

**Figure 1** Immunohistochemical staining with monoclonal antibody against DNA polymerase α. DNA polymerase α positive cells were found throughout the cancer nest (× 400).

**Figure 2** Scattergram showing the correlation between DNA polymerase α positive cell rates of endoscopic biopsy and those of resected specimens. There is good correlation between the DNA polymerase α positive cells rates obained by both methods.

**Table I** Correlation of DNa polymerase α positive cell rates and clinicopathological findings

| | *DNA polymerase α positive cells rate* | | |
|---|---|---|---|
| | *<42%* | *⩽42%* | |
| Histological type | | | |
| well differentiated | 18 | 14 | NS |
| moderately differentiated | 13 | 18 | |
| Tumour size | | | |
| <5 cm | 14 | 12 | NS |
| ⩾5 cm | 17 | 20 | |
| Serosal invasion | | | |
| negative | 24 | 23 | NS |
| positive | 7 | 9 | |
| Lymphatic invasion | | | |
| negative | 9 | 14 | NS |
| positive | 22 | 18 | |
| Venous invasion | | | |
| negative | 23 | 18 | NS |
| positive | 8 | 14 | |
| Lymph node metastasis | | | |
| negative | 16 | 18 | NS |
| positive | 15 | 14 | |
| Hepatic metastasis | | | |
| negative | 27 | 21 | $P < 0.05$ |
| positive | 4 | 11 | |
| Peritoneal metastasis | | | |
| negative | 31 | 28 | NS |
| positive | 0 | 4 | |

**Figure 3** Survival curves of patients with colorectal cancer, subdivided according to the DNA polymerase α positive cell rate.

for colorectal cancer patients (Yamaguchi *et al.*, 1990). In this study, we have examined the relation between the proliferative activity determined by use of the monoclonal antibody against DNA polymerase α and the clinical outcome of colorectal cancer patients.

The prognosis of cancer patients is largely determined by tumour mass stage, grade of malignancy, and host immunity. The grade of malignancy depends on the proliferative activity and metastatic potential of tumours. Recent reports argue that tumours with high proliferative activity have a poorer prognosis than those with low proliferative rates (Tribubait *et al.*, 1983; Sledge *et al.*, 1988; Yonemura *et al.*, 1988; Yonemura *et al.*, 1990*a*).

Lelle *et al.* (1987) argue that among 154 patients with breast cancer they examined, the proliferative activity determined using monoclonal antibody Ki-67 was significantly higher for lymph node-positive patients than for node-negative ones and, further, that this antibody is useful in predicting the prognosis and choosing an adequate therapeutic modality. Yonemura *et al.* (1990*b*) studied the proliferative activity in gastric cancer using Ki-67 monoclonal antibody and reported that the patients with high proliferative activity died significantly earlier than those with lower proliferative activity. In this study, we divided the tumours into two groups by DNA polymerase α positive cell rate and examined the relation between proliferative activity and the clinicopathologic features. Immunohistochemical staining with monoclonal antibody against DNA polymerase α offers a sensitive, simple, and objective technique for detection of growth fraction. However, DNA polymerase α immunoreactivity can be detected in only fresh material fixed in PLP or PFA. So, tissue samples should be frozen immediately and stored at −70°C. There was no correlation between the growth fractions of tumours and histological type, invasion of bowel wall, lymphatic invasion, venous invasion or lymph node metastases. However, we found a correlation between the proliferative activity determined with the monoclonal antibody against DNA polymerase α and liver metastases. This finding indicated that the determination of growth fraction by use of the monoclonal antibody against DNA polymerase α may be useful in judging the existence of liver metastasis of colorectal cancers.

DNA polymerase α positive cell rates were in close correlation with the prognosis of colorectal cancers. The prognosis was poor in colorectal cancer patients with high DNA polymerase α positive cell rate, whereas those with low DNA polymerase α positive cell rates have favourable prognosis. Colorectal cancer patients with high growth fraction tumours died significantly earlier than those with low growth fraction tumours. The univariate analysis revealed a correlation between the prognosis and the tumour size, venous invasion, lymph node metastasis, hepatic metastasis, peritoneal metastasis, and DNA polymerase α positive cells rates. However, the results of multivariate analysis using the proportional hazards model of Cox also indicated that the DNA polymerase α positive cell rate, hepatic metastasis, and peritoneal metastasis emerged as independent prognostic factors. Growth fraction was the most valuable indicator of prognosis (Table II).

In other words, it is important to know the proliferative activity pre-operatively in choosing an adequate therapeutic method and predicting the prognosis. We tried to compare the growth fractions of biopsy and resected specimens by use of a monoclonal antibody against DNA polymerase α. There is a good correlation between the DNA polymerase α positive cell rates of the two groups. Regarding intratumoural heterogeneity of growth fraction, it is possible to avoid overlooking small clones of tumour cells by taking several biopsy specimens from separate areas. We therefore came to the conclusion that it would be possible to analyse preoperatively thc proliferative activity detected in several biopsy specimens by use of a monoclonal antibody against DNA polymerase α.

From the findings, it may be concluded that the detection of growth fraction by use of a monoclonal antibody against DNA polymerase α pre-operatively enables the measurement of proliferative activity in biopsy specimens, and that the growth fraction of colorectal tumours is a useful indicator in projecting the prognosis. Tumours with a high growth fraction run poor prognoses and it is advisable, therefore, to perform intensive postoperative therapy for such tumours because the chemosensitivity of tumours is related to the ratio of proliferating cells.

**Table II** Variables of independent prognosis importance in 63 patients with colorectal cancer

| Prognostic variable | Univariate analysis Z value | Univariate analysis P value | Multivariate analysis F value | Multivariate analysis P value |
|---|---|---|---|---|
| Histological type (well moderately diff.) | 0.907 | 0.365 | 0.284 | 0.596 |
| Tumour size (<5 cm ⩾5 cm) | 2.074 | 0.038 | 0.358 | 0.552 |
| Serosal invasion (negative positive) | 2.491 | 0.013 | 1.085 | 0.302 |
| Lymphatic invasion (negative positive) | 0.4886 | 0.625 | 0.670 | 0.417 |
| Venous invasion (negative positive) | 2.660 | 0.007 | 2.477 | 0.122 |
| Lymph node metastasis (negative positive) | 2.717 | 0.007 | 3.053 | 0.086 |
| Hepatic metastasis (negative positive) | 5.105 | 0.000 | 5.334 | 0.025 |
| Peritoneal metastasis (negative positive) | 2.188 | 0.029 | 4.230 | 0.045 |
| DNA polymerases α positive cells rate (<42% ⩾42%) | 3.236 | 0.001 | 6.241 | 0.016 |

### References

BARLOGIE, B., RABER, M.N., SCHUMANN, J. & 6 others (1983). Flow cytometry in clinical cancer research. *Cancer Res.*, **43**, 3982.

BENSCH, K.G., TAKABA, S., HU, S.-Z., WANG, T.S.-F. & KORN, D. (1982). Intracellular localization of human DNA polymerase α with monoclonal antibodies. *J. Biol. Chem.*, **257**, 8391.

BLEIBERG, H. & GALANA, P. (1976). *In vivo* autoradiographic determination of cell kinetics parameters in adenocarcinomas and adjacent healthy mucosa of the colon and rectum. *Cancer Res.*, **36**, 325.

GERDES, J., SCHWAB, U., LEMKE, H. & STEIN, H. (1983). Production of a mouse monoclonal antibody reactive with a human nuclear antigen associated with cell proliferation. *Int. J. Cancer*, **31**, 13.

GERDES, J., LEMKE, H., BAISCH, H., WACKER, H.-H. (1984). Cell cycle analysis of a cell proliferation associated human nuclear antigen defined by the monoclonal antibody Ki-67. *J. Immunol.*, **13**, 1710.

GRATZNER, H.G. (1982). Monoclonal antibody to 5-bromo and 5-iodeoxy uridine. A new reagent for detection of DNA replication. *Science*, **218**, 474.

LELLE, R.J., HEIDENREICH, W., STAUCH, G. & GERDES, G. (1987). The correlation of growth fraction with histologic grading and lymph node status in human mammary carcinoma. *Cancer*, **59**, 83.

MASAKI, S., SHIKU, H., KANEDA, T., KOIWAI, O. & YOSIDA, S. (1982). Production and characterization of monoclonal antibody against 10S DNA polymerase α from calf thymus. *Nucleic Acids Res.*, **10**, 4703.

MATSUKAGE, A., YAMAMOTO, T., YAMAGUCHI, M., KUSAKABE, M., SETO, M. & TAKAHASHI, T. (1983). Immunocytochemical localization of Chick DNA polymerase α and β. *J. Cell. Physiol.*, **117**, 266.

MUSHIKA, M., MIWA, T., SUZUKI, Y., HAYASHI, K., MASAKI, S. & KANEDA, T. (1988). Detection of proliferating cells in dysplasia, carcinoma *in situ*, and invasive carcinoma of uterine cervix by monoclonal antibody against DNA polymerase α. *Cancer*, **61**, 1182.

NAKAMURA, H., MORITA, T., MASAKI, S. & YOSHIDA, S. (1984). Intracellular localization and metabolism of DNA polymerase α in human cells visualized with monoclonal antibody. *Exp. Cell Res.*, **151**, 123.

SARNGADHARAN, M.G., ROBERT-GUROFF, M. & GALLO, R. (1978). DNA polymerases of normal and neoplastic mammalian cells. *Biochem. Biophys. Acta.*, **516**, 419.

SASAKI, K. (1977). Measurement of tritiated thymidine labeling index by incubation *in vitro* of surgically removed cervical cancer. *Gann*, **68**, 307.

SCHUTTE, B., REYNDERS, M.M., WIGGER, T. & 4 others (1987). Retrospective analysis of the prognostic significance of DNA content and proliferative activity in large bowel carcinoma. *Cancer Res.*, **47**, 5494.

SLEDGE, G.W., EBLE, J.N., ROTH, B.J., WUHRMAN, B.P., FINEBERG, N. & EINHORN, L.H. (1988). Relation of proliferative activity in patients with advanced germ cell tumor. *Cancer Res.*, **48**, 3864.

TRIBUBAIT, B., HAMMERBERG, C. & RUBIO, C. (1983). Ploidy and proliferation patterns in colorectal adenocarcinomas related to Dukes' classification and to histopathological differentiation. *Acta Pathol. Microbiol. Immunol. Second. (Sect. A)*, **91**, 89.

VOLM, M., HAHN, E.W., MATTERN, J. & 3 others (1988). Five-year follow-up study of independent clinical and flow cytometric prognostic factors for the survival of patients with non-small cell lung carcinoma. *Cancer Res.*, **48**, 2923.

WEISSBACH, A. (1979). The functional roles of mammalian DNA polymerase. *Arch. Biochem. Biophys.*, **198**, 386.

YAMAGUCHI, A., TAKEGAWA, S., ISHIDA, T. & 6 others (1990). Detection of the growth fraction in colorectal tumours by a monoclonal antibody against DNA polymerase α. *Br. J. Cancer*, **61**, 390.

YONEMURA, Y., SUGIYAMA, K., FUJIMURA, T. & 6 others (1988). Correlation of DNA ploidy and proliferative activity in human gastric cancer. *Cancer*, **62**, 1497.

YONEMURA, Y., OOYAMA, S., SUGIYAMA, K. & 7 others (1990). Retrospective analysis of the significance of DNA ploidy patterns and S-phase fraction in gastric carcinoma. *Cancer Res.*, **50**, 509.

YONEMURA, Y., OHOYAMA, S., KIMURA, H. & 6 others (1990). Assessment of tumor cell kinetics by monoclonal antibody Ki-67. *Eur. Surg. Res.*, **22**, 365.